# THBS1 Induces Dysfunction of Ovarian Granulosa Cells in Patients with Polycystic Ovary Syndrome by Activating the TGF-β/Smad Pathway

**DOI:** 10.3390/biomedicines14061273

**Published:** 2026-06-02

**Authors:** Jiajing He, Lirong Wang, Luni Tan, Xinyue Zhou, Xiaorong Luo, Wei Wang, Xuehong Zhang

**Affiliations:** 1 The First Clinical Medical College, Lanzhou University, Lanzhou 730000, China; hejj626488@126.com (J.H.); 13893418910@163.com (L.W.); tln1999tln@163.com (L.T.); 120240902990@lzu.edu.cn (X.Z.); luoxr2023@lzu.edu.cn (X.L.); wangwei83819@163.com (W.W.); 2 The Reproductive Medicine Center, The First Hospital of Lanzhou University, Lanzhou 730000, China

**Keywords:** polycystic ovary syndrome, THBS1, TGF-β1, Smad2, granulosa cell apoptosis

## Abstract

**Objective:** This study aims to investigate the role of thrombospondin-1 (THBS1) in polycystic ovary syndrome (PCOS) pathogenesis and its mechanism in regulating granulosa cell (GC) function. **Methods:** Follicular fluid and granulosa cells from 21 PCOS patients and 21 age-matched non-PCOS controls were analysed for THBS1 expression and clinical correlations. A dehydroepiandrosterone (DHEA)-induced PCOS rat model with adeno-associated virus serotype 9 (AAV9)-mediated THBS1 knockdown was used to assess phenotypic changes. The KGN human granulosa-like cell line was employed to evaluate THBS1 overexpression effects on proliferation, apoptosis, and steroidogenesis. Mechanistic studies included RNA sequencing with Gene Set Enrichment Analysis (GSEA), co-immunoprecipitation, molecular docking against the latent TGF-β1 crystal structure (PDB 9VJJ), molecular dynamics simulation, an active/total TGF-β1 ELISA, and pharmacological TGF-β receptor inhibition. **Results:** THBS1 was elevated in PCOS follicular fluid and granulosa cells and correlated positively with serum AMH and LH after Benjamini–Hochberg FDR correction. AAV9-mediated ovarian THBS1 knockdown (37.4% protein reduction, *p* = 0.006) ameliorated cystic morphology, restored estrous cyclicity, and normalised serum AMH/LH/T. In KGN cells, THBS1 overexpression suppressed proliferation, induced apoptosis and inflammatory cytokines, and dysregulated steroidogenic enzymes. Transcriptome analysis revealed upregulation of canonical TGF-β/Smad pathway components (SERPINE1, SMAD7, TGFB2, INHBA, CCN2, COL1A1/2). Molecular docking and 100-ns dynamics simulation supported a stable interaction between THBS1 and latent TGF-β1 (ΔG_TOTAL ≈ −120 kcal·mol^−1^). Co-immunoprecipitation confirmed physical association in cells, and ELISA showed elevated TGF-β1 in PCOS follicular fluid and rat serum, both attenuated by THBS1 knockdown. Pharmacological TGF-β receptor inhibition with SB-431542 rescued THBS1-induced cellular dysfunction. **Conclusions:** THBS1 is associated with PCOS-related granulosa cell dysfunction through the TGF-β/Smad pathway and represents a candidate biomarker and exploratory therapeutic target that warrants validation in independent multicentre cohorts.

## 1. Introduction

Polycystic ovary syndrome (PCOS) is one of the most common endocrine and metabolic disorders affecting women of reproductive age, with global prevalence rates ranging from 7.8% to 12.7% depending on the diagnostic criteria used [1]. It is characterized by heterogeneous manifestations, including hyperandrogenism, ovulatory dysfunction, polycystic ovarian morphology, and metabolic abnormalities such as insulin resistance, type 2 diabetes, metabolic syndrome, and cardiovascular diseases, which severely affect female fertility and long-term health [2,3]. However, the etiology and pathophysiology of PCOS remain unclear, presenting challenges for diagnosis and treatment [4]. Therefore, elucidating its pathogenesis is essential for the development of novel diagnostic markers and exploratory therapeutic candidates.

Ovarian granulosa cells (GCs), which serve as the primary functional units for follicular development and steroidogenesis, play a pivotal role in the pathophysiology of PCOS [5]. Dysregulation of their proliferation, apoptosis, and function constitutes a central event in ovarian dysfunction associated with PCOS. This imbalance not only disrupts the selection and dominance of the leading follicle, resulting in anovulation, but also exacerbates systemic endocrine disturbances by altering local hormone synthesis and the homeostasis of the follicular microenvironment [6]. Recent studies emphasize the importance of inflammatory responses and aberrant signaling pathways in regulating GC function [7]. Among these, the Transforming Growth Factor-β(TGF-β)/Smad signaling pathway is a key regulator of GC proliferation, differentiation, and steroidogenesis. Dysregulated activation or inhibition of this pathway is closely implicated in the pathogenesis of PCOS [8,9,10]. TGF-β signaling is initiated when ligands bind to membrane receptors, leading to the phosphorylation of downstream Smad2/3 proteins, which then translocate to the nucleus to regulate target gene transcription and, ultimately, influence GC function. However, the upstream molecular triggers responsible for the abnormal activation of this pathway in PCOS remain unclear.

Thrombospondin-1 (THBS1) is a multifunctional extracellular matrix (ECM) glycoprotein involved in cell adhesion, proliferation, apoptosis, and inflammatory responses [11,12,13]. Emerging evidence indicates that THBS1 is abnormally expressed in various metabolic and inflammation-related disorders. Notably, it can regulate the activity of TGF-β family members through direct interaction, thereby influencing pathway activation. Previous studies have demonstrated that THBS1 expression is significantly elevated in granulosa cells and ovarian tissues of both PCOS patients and animal models [14,15,16,17]. Additionally, THBS1 has been proposed as a potential biomarker for ovarian hyperstimulation syndrome and premature ovarian insufficiency [18,19,20]. However, the expression level and function of THBS1 in the ovarian microenvironment of PCOS, as well as its relationship with the TGF-β pathway, remain unclear. Moreover, its role in GC dysfunction is an unexplored area.

Based on this, we hypothesize that locally elevated THBS1 in the ovaries of PCOS patients excessively activates the TGF-β/Smad signaling pathway. This activation, in turn, induces GC dysfunction characterized by suppressed proliferation, increased apoptosis, inflammatory response, and disrupted steroid hormone synthesis, thereby contributing to the anovulation and endocrine imbalance observed in PCOS. To test this hypothesis, our study integrates a multi-tiered experimental approach encompassing clinical sample analysis, animal model validation, cellular functional assays, and molecular mechanism exploration. First, we compared THBS1 levels in follicular fluid from PCOS patients and normal controls and analyzed their correlation with key clinical parameters (clinical baseline characteristics are shown in Table 1). Second, utilizing a dehydroepiandrosterone (DHEA)-induced PCOS rat model, we investigated the phenotypic improvements following in vivo knockdown of THBS1. Subsequently, we employed the human ovarian granulosa cell line KGN to delineate the functional consequences of THBS1 overexpression. Finally, we combined transcriptomic sequencing, co-immunoprecipitation (Co-IP), molecular docking, and pathway inhibitor interventions to elucidate the core molecular mechanism whereby THBS1 directly interacts with and activates the TGF-β/Smad pathway. This study aims to identify THBS1 as a novel regulatory factor in PCOS-related ovarian dysfunction, providing fresh theoretical and experimental insights for understanding PCOS pathology and developing potential exploratory therapeutic candidates.

## 2. Results

### 2.1. THBS1 Is Upregulated in PCOS Patients and Correlates with Clinical Parameters

The clinical baseline data of 21 patients with PCOS and 21 non-PCOS controls were collected and compared. The results demonstrated that significant differences were observed between the PCOS group and the control group in terms of antral follicle count (AFC), anti-Müllerian hormone (AMH), luteinizing hormone (LH), testosterone (T), homeostatic model assessment of insulin resistance (HOMA-IR) levels, total gonadotropin (Gn) dosage, serum E2 levels on the day of human chorionic gonadotropin (HCG) administration, number of follicles exceeding 16 mm on HCG day, and number of retrieved metaphase II (MII) oocytes (Table 1).

**Figure 1:**
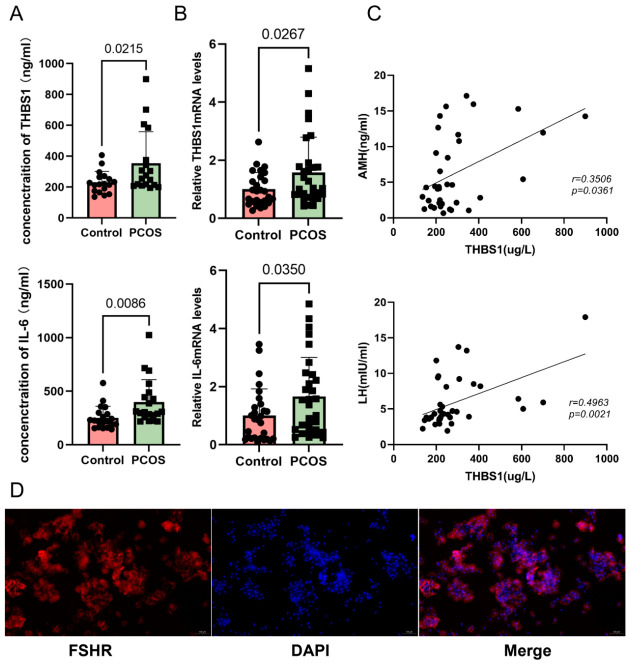
THBS1 is up-regulated in the follicular fluid and granulosa cells of PCOS patients and correlates with anti-Müllerian hormone and luteinising hormone. (**A**) Concentrations of THBS1 and IL-6 in follicular fluid from control and PCOS patients were measured by ELISA. individual data points are shown as symbols. (**B**) Relative mRNA expression levels of THBS1 and IL-6 in granulosa cells from control and PCOS patients were determined by qPCR. (**C**) Correlation analyses between follicular fluid THBS1 concentrations and serum AMH and LH levels in PCOS patients. (**D**) Immunofluorescence staining of granulosa cells for FSHR (red) and DAPI (blue). The merged image confirms granulosa cell identity. Scale bar = 50 μm.

To investigate the potential role of THBS1 in PCOS pathogenesis, we first examined the expression levels of THBS1 and IL-6 in follicular fluid and granulosa cells from PCOS patients and normal control (NC) subjects. As shown in Figure 1A, ELISA analysis revealed that the concentration of THBS1 in follicular fluid was significantly elevated in PCOS patients compared to controls (*p* = 0.0215), accompanied by increased IL-6 levels (*p* = 0.0086). Consistently, quantitative PCR analysis demonstrated that the mRNA expression levels of both THBS1 and IL-6 were significantly upregulated in granulosa cells derived from PCOS patients relative to control subjects (THBS1: *p* = 0.0267; IL-6: *p* = 0.0350) (Figure 1B). To further explore the clinical relevance of elevated THBS1 levels in PCOS, we performed correlation analyses between follicular fluid THBS1 concentrations and key clinical parameters. Notably, THBS1 levels showed a significant positive correlation with anti-Müllerian hormone (AMH) levels (r = 0.3506, *p* = 0.0361) and luteinizing hormone (LH) levels (r = 0.4963, *p* = 0.0021) (Figure 1C), suggesting that THBS1 may be associated with the severity of PCOS-related endocrine dysfunction. Finally, to confirm the purity of isolated granulosa cells used in this study, we performed immunofluorescence staining for follicle-stimulating hormone receptor (FSHR), a specific marker for granulosa cells. The results showed positive FSHR staining (red) with clear nuclear counterstaining by DAPI (blue), and the merged image confirmed the identity of granulosa cells (Figure 1D). Collectively, these findings indicate that THBS1 is aberrantly upregulated in both follicular fluid and granulosa cells of PCOS patients and correlates positively with AMH and LH levels, implicating THBS1 as a potential biomarker and exploratory therapeutic candidate in PCOS.

### 2.2. Knockdown of Thbs1 Ameliorates the PCOS-like Phenotype in a Rat Model

**Figure 2:**
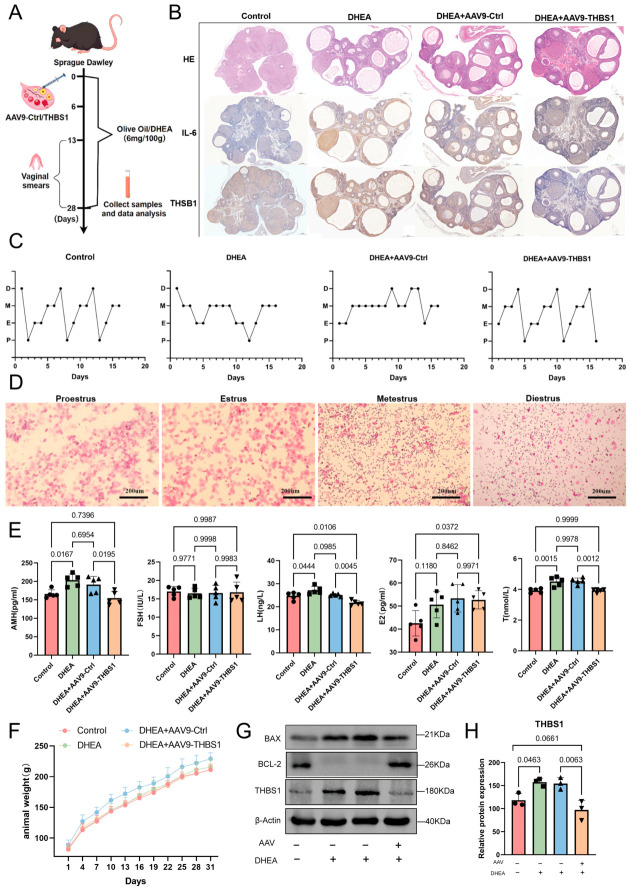
AAV9-mediated knockdown of ovarian Thbs1 ameliorates the PCOS-like reproductive phenotype in a DHEA-induced rat model. (**A**) The timeline schematic was created using FigDraw (https://www.figdraw.com), illustrating DHEA-induced PCOS establishment and AAV9-mediated THBS1 knockdown intervention. (**B**) Representative HE staining and immunohistochemistry images of ovarian sections. Scale bar = 500 μm. (**C**) Quantification of time spent in each estrous cycle stage. (**D**) Vaginal smear cytology showing estrous cycle stages. Scale bar = 200 μm. (**E**) Serum hormone levels (AMH, FSH, LH, E2, T) measured by ELISA. Different colored bars represent different experimental groups, different symbols indicate individual animal data point. (**F**) Body weight changes during the 28-day protocol. (**G**) Western blot analysis of ovarian THBS1, BAX, and BCL-2 protein expression. (**H**) AAV9-mediated ovarian THBS1 knockdown (37.4% protein reduction, *p* = 0.006), band intensity was calculated by Image J.

To investigate the functional role of THBS1 in the pathogenesis of PCOS, we employed an AAV9-mediated knockdown approach in a classic DHEA-induced rat model of PCOS. The timeline for PCOS induction and subsequent interventions is shown in (Figure 2A). Ovarian morphology was assessed by HE staining and immunohistochemistry (Figure 2B). DHEA-induced PCOS rats exhibited multiple cystic follicles, reduced corpora lutea, and increased ovarian THBS1 and IL-6 expression. AAV9-Ctrl treatment did not alter these effects, whereas AAV9-THBS1 treatment significantly ameliorated ovarian morphology, accompanied by reduced THBS1 and IL-6 expression, suggesting alleviation of cystic degeneration and inflammatory response. Vaginal smear cytology (Figure 2C) showed that control rats maintained regular estrous cycles, whereas DHEA-treated rats exhibited persistent diestrus. This disruption persisted in the DHEA + AAV9-Ctrl group but was reversed in the DHEA + AAV9-THBS1 group, which showed a significantly higher percentage of time spent in proestrus and metestrus (Figure 2D), indicating restoration of cyclic ovarian activity. Hormonal profiling by ELISA (Figure 2E) revealed that DHEA induction significantly increased serum levels of AMH, LH, and T. These elevations were markedly reversed in the DHEA + AAV9-THBS1 group, with levels approaching those of the control group. Regarding metabolic changes (Figure 2F), the DHEA and DHEA + AAV9-Ctrl groups exhibited significantly greater weight gain over the 28-day protocol. Notably, THBS1 knockdown attenuated this weight gain. Finally, Western blot analysis (Figure 2G,H) confirmed that DHEA increased ovarian THBS1 expression, which was effectively abrogated by AAV9-THBS1 (37.4% protein reduction, *p* = 0.006). Furthermore, DHEA induced a pro-apoptotic imbalance (increased BAX, decreased BCL-2) (Figure 2G), which was reversed following THBS1 knockdown, suggesting that inhibition of ovarian cell apoptosis may partly mediate its protective effects.

### 2.3. Overexpression of THBS1 Induces Apoptosis and Inhibits Proliferation in KGN Cells

**Figure 3:**
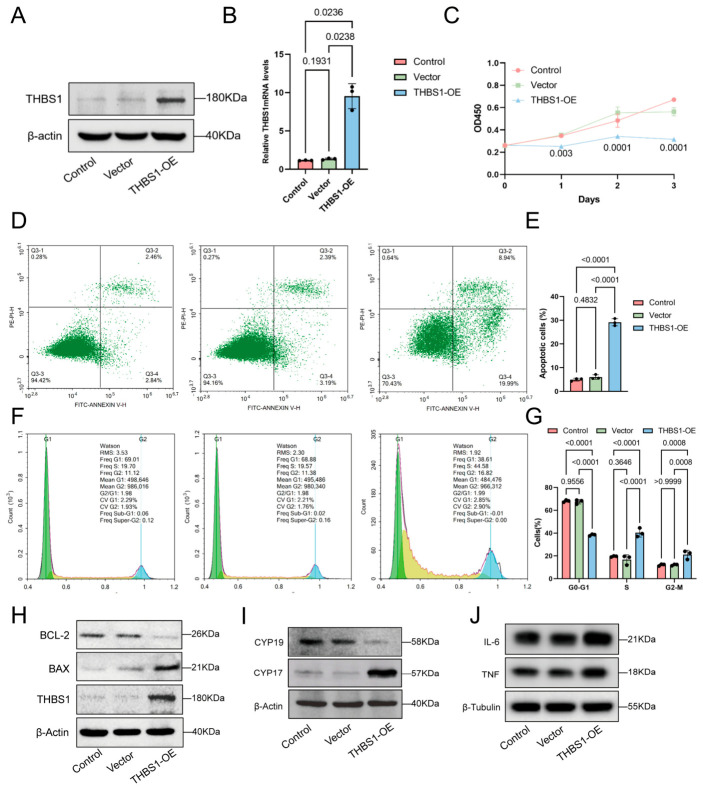
THBS1 overexpression impairs KGN Cells function. (**A**,**B**) Validation of THBS1 overexpression by Western blot and qPCR. (**C**) CCK-8 assay showing suppressed cell proliferation over 3 days. (**D**,**E**) Flow cytometry analysis of apoptosis. (**F**,**G**) Flow cytometry analysis of cell cycle distribution. (**H**–**J**) Western blot analysis of apoptosis markers (BAX, BCL-2), inflammatory cytokines (IL-6, TNF-α), steroidogenic enzymes (CYP19, CYP17).

To investigate the functional role of THBS1 in granulosa cells, we established a THBS1 overexpression (THBS1-OE) system in KGN cells. Successful overexpression was confirmed by Western blot and qPCR, showing markedly elevated THBS1 protein and mRNA levels compared to control groups (Figure 3A,B). CCK-8 assay demonstrated that THBS1 overexpression significantly suppressed KGN cell proliferation over 3 days (Figure 3C). THBS1 overexpression significantly induces granulosa cell apoptosis (*p* < 0.0001) while blocking cell cycle progression, manifested by decreased G0/G1 phase proportion and increased S phase proportion (both *p* < 0.0001), suggesting that THBS1 may impair granulosa cell function by promoting G2/M or S phase arrest and triggering apoptotic cell death (Figure 3D–G). Western blot analysis further showed that THBS1 overexpression altered the expression of apoptosis-related proteins (BAX upregulation and BCL-2 downregulation), elevated inflammatory cytokines (IL-6 and TNF-α), and dysregulated steroidogenic enzymes (CYP19 and CYP17) (Figure 3H,I). Collectively, these findings indicate that THBS1 overexpression impairs granulosa cell function through multiple mechanisms including enhanced apoptosis, cell cycle arrest, and dysregulation of inflammatory and steroidogenic pathways.

### 2.4. Molecular Docking and Dynamics Simulation Support a Stable THBS1–Latent-TGF-β1 Interaction

**Figure 4:**
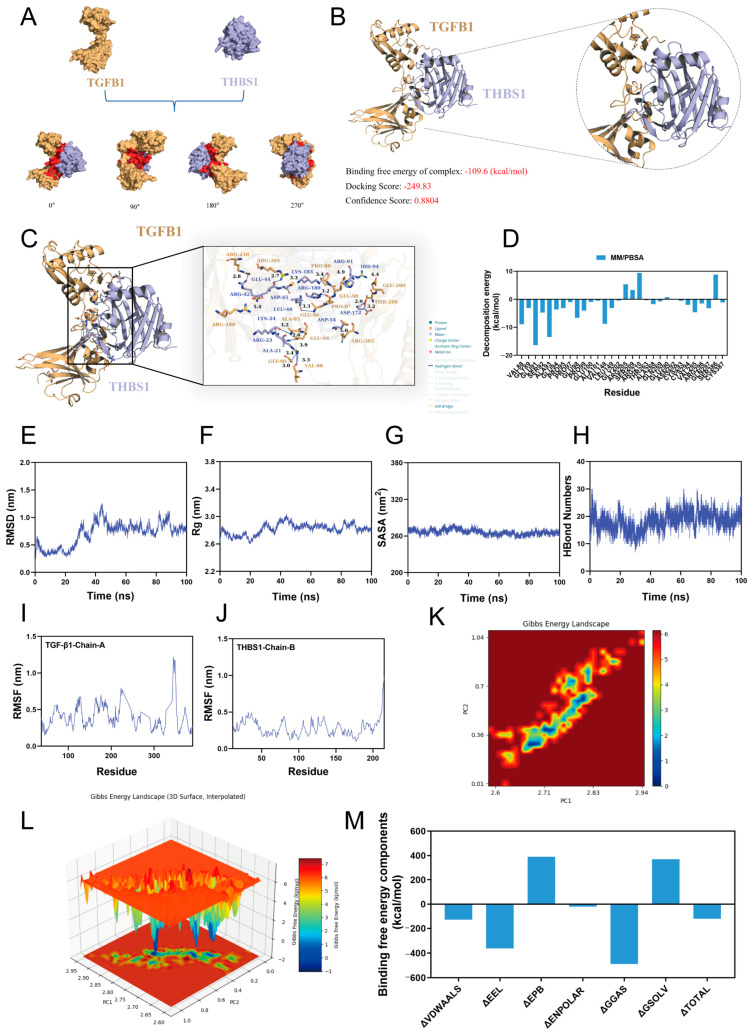
Molecular docking and 100-ns dynamics simulation reveal a stable association between THBS1 and human latent TGF-1 (PDB 9VJJ). (**A**) Surface representation of THBS1-latent TGF-β1 (PDB 9VJJ) binding interface. Brownish-yellow: TGF-β1, purple-blue: THBS1, red: interfacial binding residues. (**B**) Docking scores and confidence scores of top models. (**C**) Detailed interaction analysis showing hydrogen bonds (blue lines) and salt bridges (yellow dashed lines) between THBS1 (blue) and latent TGF-β1 (PDB 9VJJ) (yellow). (**D**) Per-residue MM/PBSA decomposition. (**E**) RMSD analysis of the protein complex over 100 ns. (**F**) Radius of gyration (Rg) analysis showing structural compactness. (**G**) Solvent-accessible surface area (SASA) analysis. (**H**) Hydrogen bond count throughout the simulation. RMSF analysis of THBS1 (**I**) and latent TGF-β1 (PDB 9VJJ) (**J**) residues. (**K**,**L**) Gibbs free-energy landscape (FEL) based on RMSD and Rg. (**M**) MM/PBSA binding free energy decomposition.

To investigate the THBS1–TGF-β1 interaction at the atomic level, the structure of human latent TGF-β1 (PDB 9VJJ, deposited June 2025; Mol_ID 1, chains A and B comprising the latency-associated peptide (LAP) and the mature TGF-β1 cytokine) was retrieved from the Protein Data Bank. The co-crystallised SOF10 Fab (chains H, I, L and M) was removed prior to docking, and the THBS1 receptor model was downloaded from UniProt (entry P07996) and energy-minimised. Protein–protein docking was performed using HDOCKlite v1.1 with default sampling and template-free settings, and the binding interface was visualised using PyMOL (Schrödinger, v2.5). The top-ranked complex showed a docking score of −262.32 with a confidence score of 0.9043, indicating high likelihood of interaction (Figure 4A,B).

PLIP-based interaction analysis identified a dense interface comprising both polar and hydrophobic contacts between THBS1 (chain B) and latent TGF-β1 (chain A) (Figure 4C). Hydrogen bonds at distances between 2.6 and 3.4 Å were observed for the residue pairs ARG-205↔LYS-183 (2.7 Å), GLU-44↔ARG-180 (3.3 Å), ASP-45↔ARG-180 (3.4 Å), GLU-94↔ASP-54 (2.6 Å), GLU-98↔ARG-91 (3.3 Å), PRO-99↔ARG-91 (4.9 Å), and PRO-97↔ASP-172 (3.4 Å). Salt bridges between ARG-385↔GLU-296 (5.0 Å), HIS-94↔GLU-290 (4.4 Å) and ARG-42↔ASP-45 (5.4 Å) stabilised the interface (Figure 4C). Per-residue MM/PBSA decomposition (Figure 4D) further confirmed the dominant contributions of GLU-91 (−16.6 kcal·mol^−1^), ALA-93 (−13.3), VAL-88 (−9.0), VAL-148 (−8.7) and SER-92 (−4.8) to binding.

To assess the stability of the predicted complex, three independent 100-ns molecular dynamics (MD) simulations were performed in GROMACS 2023.2 with the AMBER14SB protein force field; representative trajectories are shown. The Cα root-mean-square deviation (RMSD) of the complex equilibrated near 0.7–0.9 nm after approximately 40 ns and remained stable throughout the remainder of the trajectory (Figure 4E). The radius of gyration (Rg) fluctuated between 2.6 and 3.0 nm, indicating that overall structural compactness was preserved (Figure 4F). The solvent-accessible surface area (SASA) of the complex remained between 260 and 280 nm^2^ with only minor fluctuation (Figure 4G), consistent with a stable intermolecular interface. The number of intermolecular hydrogen bonds varied between 12 and 28 with a mean of approximately 18 (Figure 4H), and per-residue root-mean-square fluctuation (RMSF) showed that the majority of residues in both chains exhibited low flexibility (<0.5 nm), with elevated motion confined to terminal loops (Figure 4I,J). The Gibbs free-energy landscape constructed from the first two principal components revealed a single well-defined energy minimum, indicating that the complex adopted a single dominant conformational state (Figure 4K,L).

Quantitative MM/PBSA analysis estimated a total binding free energy of ΔG_TOTAL ≈ −120 kcal·mol^−1^, with electrostatic interactions (ΔEEL = −360 kcal·mol^−1^) and van der Waals contacts (ΔVDW = −130 kcal·mol^−1^) as the major favourable terms, partially offset by polar solvation (ΔEPB = +390 kcal·mol^−1^) (Figure 4M). Together, these computational data support a stable, thermodynamically favourable association between THBS1 and the latent TGF-β1 proprotein. They are consistent with the biochemical interaction shown in Section 2.5, although they do not by themselves prove it.

### 2.5. RNA-Seq and Co-Immunoprecipitation Implicate THBS1 in TGF-β/Smad Pathway Activation

We performed RNA sequencing of KGN cells stably expressing THBS1 (THBS1-OE, n = 3) versus empty-vector controls (Control, n = 3). Sample identity was verified by THBS1 expression and corroborated by in-house qPCR before downstream analysis; one sample-tube labelling discrepancy was identified post-sequencing and corrected in the final dataset (see Section 4.5).

**Figure 5:**
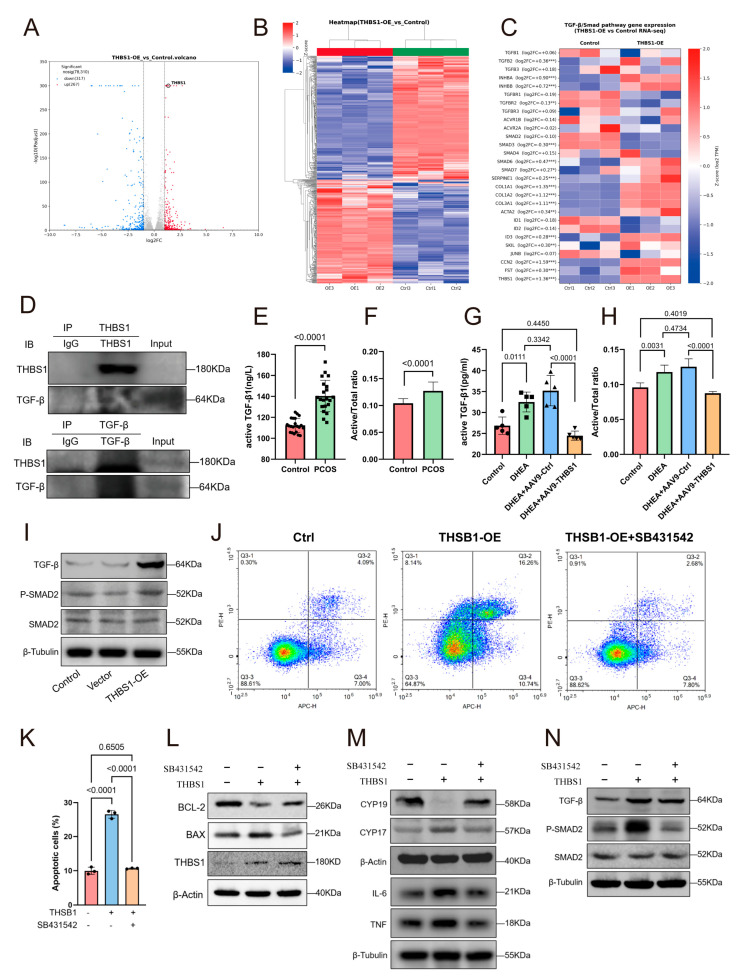
THBS1 overexpression activates the TGF-β1/Smad axis. (**A**) DESeq2 volcano (267 up + 317 down at |log FC| > 1, Padj < 0.05). (**B**) Hierarchical clustering of differentially expressed genes. (**C**) TGF-β/Smad pathway gene expression (28 canonical genes). *, **, *** Data are presented as mean ± SEM. (**D**) Reciprocal co-immunoprecipitation: lanes IgG/IP/Input. (**E**) Active TGF-β1—patient FF. Different colored bars represent different experimental groups, and different symbols indicate individual sample data points. (**F**) Active/Total ratio--patient FF. (**G**) Active TGF-β1—rat serum. (**H**) Active/Total ratio—rat serum. (**I**) Western blot analysis of TGF-β/Smad—KGN cells (THBS1-OE). (**J**,**K**) Flow cytometry apoptosis (THBS1-OE ± SB-431542). (**L**–**N**) Western blot analysis of apoptosis markers (BAX, BCL-2), inflammatory cytokines (IL-6, TNF-α), steroidogenic enzymes (CYP19, CYP17), and TGF-β/SMAD2 signaling pathway components.

After alignment with HISAT2 v2.2.1 and quantification by RSEM v1.3.3, differential expression analysis with DESeq2 v1.42.0 (|log_2_FC| > 1, BH-adjusted *p* < 0.05) identified 584 differentially expressed genes (DEGs) between THBS1-OE and Control samples (267 up-regulated, 317 down-regulated). THBS1 itself was the strongest single up-regulated DEG (log_2_FC = +1.36, BH-adjusted *p* < 1 × 10^−150^), confirming successful overexpression at the transcript level (Figure 5A,B; Supplementary Figures S1 and S2 for PCA and sample-correlation diagnostics). Over-representation analysis of the up-regulated DEGs identified ECM–receptor interaction as the most significantly enriched pathway (8/88 genes; BH-adjusted *p* = 0.002), containing THBS1 itself, COL1A1/2, COL3A1 and SPP1, consistent with THBS1’s established role as a matricellular ECM glycoprotein. Down-regulated DEGs were dominated by interferon and antiviral response pathways (Influenza A, Epstein–Barr virus infection, NOD-like receptor signalling; BH-adjusted *p* < 1 × 10^−6^ each).

Although the TGF-β signalling pathway as a whole did not reach significance by over-representation analysis, GSEA on the rank-ordered genome-wide expression list revealed positive enrichment for the canonical KEGG TGF-β signalling pathway in THBS1-OE cells (NES = +1.45, nominal *p* = 0.007; Reactome “TGF-β receptor signalling activates SMADs” NES = +1.33, nominal *p* = 0.081). At the gene level, multiple canonical TGF-β/Smad pathway components were significantly altered in THBS1-OE cells: the direct Smad target CCN2 (CTGF) was strongly up-regulated (log_2_FC = +1.59, BH-adjusted *p* < 1 × 10^−150^), as were the matricellular targets COL1A1 (+1.35), COL1A2 (+1.12) and COL3A1 (+1.11). The TGF-β-family ligands TGFB2 (+0.36, *p* = 1.9 × 10^−9^) and INHBA (+0.90, *p* = 2.6 × 10^−33^), the inhibitor FST (+0.30, *p* = 1.5 × 10^−5^), and the negative-feedback regulator SMAD7 (+0.27, *p* = 0.013) were also elevated together. These transcriptional signatures (Figure 5C) provide a transcriptome-wide line of evidence for TGF-β/Smad pathway activation by THBS1 overexpression.

To test whether THBS1 physically associates with TGF-β1 in cells, we performed reciprocal co-immunoprecipitation in KGN extracts. Each blot in Figure 5D contains three lanes loaded as: lane 1, isotype-matched IgG (negative control); lane 2, immunoprecipitate with the specific antibody (IP); lane 3, 5% input (whole-cell lysate). Immunoprecipitation with anti-THBS1 antibody (Proteintech 18304-1-AP, lane 2) co-precipitated TGF-β1 detectable above the IgG-control band, and reciprocally immunoprecipitation with anti-TGF-β1 (Proteintech 10188-1-AP, lane 2) co-precipitated THBS1, while the IgG-control lane in each blot showed no specific signal (Figure 5D).

We next asked whether THBS1 promotes activation of latent TGF-β1. Active and total TGF-β1 levels were quantified by ELISA (Fankw, F-series) in patient follicular fluid (Control n = 21, PCOS n = 21) and in matched rat serum from the four animal groups (n = 5 per group). The active fraction of TGF-β1 (active/total ratio) was significantly elevated in PCOS follicular fluid compared to controls (*p* < 0.0001, Figure 5E,F) and was reduced in DHEA + AAV9-THBS1 rats relative to DHEA + AAV9-Ctrl (*p* < 0.0001, Figure 5G,H), Additionally, THBS1-OE activated the TGF-β signaling pathway, as evidenced by increased phosphorylation of SMAD2 (Figure 5I), consistent with a model in which elevated THBS1 promotes activation of locally available latent TGF-β1.

Finally, to determine whether the cellular dysfunction induced by THBS1 overexpression depends on the TGF-β receptor, we treated THBS1-OE KGN cells with the type-I receptor (ALK4/5/7) inhibitor SB-431542 (MedChemExpress HY-10431, 10 μM, 48 h). Flow-cytometric apoptosis analysis showed that SB-431542 significantly reduced the apoptotic fraction in THBS1-OE cells (*p* < 0.0001, versus untreated THBS1-OE), restoring it close to control levels (*p* = 0.65 versus Control) (Figure 5J,K). Western blot demonstrated that SB-431542 attenuated the THBS1-induced increase in BAX, IL-6, TNF-α and CYP17A1; restored BCL-2 and CYP19A1; and abrogated phosphorylation of SMAD2/3 (Figure 5L–N). Collectively, these findings support a model in which THBS1 promotes TGF-β1 activation, and the resulting TGF-β1/TGFβR1/Smad axis mediates the granulosa cell phenotype observed in PCOS-relevant settings.

## 3. Discussion

PCOS is a highly heterogeneous disorder with diverse phenotypes and clinical manifestations, presenting numerous uncertainties in its pathophysiology, diagnosis, and management [21]. Although significant progress has been made in understanding the systemic pathophysiology of PCOS, the local regulatory mechanisms within the ovary remain poorly understood. The follicular microenvironment, composed of follicular fluid and ovarian granulosa cells, plays a key role in folliculogenesis, oocyte maturation, and steroid hormone synthesis [6]. Abnormalities in follicular fluid composition can directly disrupt GC function, leading to follicular developmental arrest and ovulatory dysfunction, which are core pathological features of PCOS [22,23,24]. Consequently, identifying key regulatory molecules within the follicular microenvironment and exploring their roles in GC dysfunction are essential for understanding the local pathogenesis of PCOS and developing targeted therapeutic strategies.

Thrombospondin-1 (THBS1) is a multifunctional ECM protein that exerts regulatory effects at the interface between the ECM and the cell surface [25]. Recent studies have expanded the recognized functions of THBS1 from regulating cell adhesion and angiogenesis to modulating inflammation, responses to hypoxia and genotoxic stress, redox signaling, stem cell self-renewal, and autophagy. These functions are mediated through interactions with cell surface receptors, structural components of the ECM, and secreted factors [11,26]. As a multifunctional molecule linking inflammation, the extracellular matrix, and signal transduction, THBS1 is a candidate for in-depth investigation. In PCOS, THBS1 expression is significantly elevated, suggesting its potential involvement in the pathological process [15,16]. Our study confirmed that elevated THBS1 levels in the follicular fluid and granulosa cells of PCOS patients were accompanied by a concurrent increase in the inflammatory cytokine IL-6. This finding implies a close association between THBS1 and the disturbed inflammatory microenvironment in PCOS. Correlation analysis further revealed positive relationships between THBS1 levels and serum concentrations of anti-Müllerian hormone (AMH) and luteinizing hormone (LH). Given that AMH is a key marker of ovarian reserve and polycystic morphology [27,28], and elevated LH is a central PCOS phenotypes [29], these results suggest that abnormal expression of THBS1 is closely linked to core pathological features of PCOS, highlighting its potential as a novel biomarker for the syndrome.

To further validate the functional role of THBS1, a DHEA-induced PCOS rat model was employed. This classic model recapitulates core human PCOS phenotypes, including disrupted estrous cyclicity, abnormal weight gain, sex hormone imbalance, and ovarian morphological changes [30,31]. Our results showed that DHEA induction led to typical PCOS phenotypes, along with a significant increase in ovarian THBS1 expression. Importantly, AAV-mediated THBS1 knockdown ameliorated the disrupted estrous cycles, hormonal imbalance, and ovarian cell apoptosis in these rats. These in vivo findings confirm that aberrantly elevated THBS1 expression is a key molecular driver of PCOS-related phenotypes and demonstrate that targeted knockdown of THBS1 effectively ameliorates multiple pathological features, thereby providing an experimental foundation for THBS1-targeted therapeutic strategies for PCOS.

Ovarian granulosa cells are central to follicular development, and abnormalities in their proliferation, apoptosis, and steroidogenic function directly contribute to follicular arrest and anovulation, which constitute the core cellular basis of PCOS [32]. In vitro experiments using the human KGN granulosa cell line demonstrated that THBS1 overexpression significantly inhibited cell proliferation, promoted apoptosis, upregulated the expression of inflammatory factors IL-6 and TNF-α, and altered the expression of steroidogenic genes CYP17A1 and CYP19A1. These results support at the cellular level for the regulatory impact of THBS1 on granulosa cell function. The modulation of CYP17A1 and CYP19A1 by THBS1 further underscores its role in disrupting steroidogenic balance.

The TGF-β/Smad signaling pathway is a critical intracellular signal transduction cascade that is broadly involved in biological processes such as cell proliferation, differentiation, apoptosis, and fibrosis. Previous studies indicate that this pathway plays a significant role in granulosa cell dysfunction and PCOS pathology, influencing follicular development and GC physiology by regulating apoptosis and ECM remodeling. While physiological TGF-β signaling is essential for normal follicular growth and GC differentiation, its excessive activation can lead to growth suppression and a pro-fibrotic phenotype. Our data indicate that THBS1 overexpression in granulosa cells induces a phenotype characteristic of TGF-β pathway hyperactivation, including suppressed proliferation inhibition (likely via cell cycle arrest), increased apoptosis (potentially through upregulation of pro-apoptotic proteins), and enhanced release of pro-inflammatory factors (IL-6, TNF-α). This finding offers a novel mechanistic perspective for explaining the developmental arrest of dominant follicles and the chronic low-grade inflammatory state in PCOS ovaries. Notably, the interference of THBS1 with key steroidogenic enzymes (CYP17A1, CYP19A1) may directly perturb the estrogen/androgen balance, thereby partially accounting for the local hyperandrogenic microenvironment in PCOS.

THBS1 is known to promote activation of latent TGF-β, converting it to its active form and thereby initiating downstream Smad-dependent signaling. For instance, BMP-1-mediated cleavage of THBS1 enhances its ability to activate latent TGF-β, thereby augmenting TGF-β/Smad signaling and regulating processes such as cell adhesion and fibrosis [33]. Another study demonstrated that THBS1 enhances TGF-β1-induced Smad3 signaling, thereby increasing the expression of the fibrotic markers COL1A2 and α-SMA [34]. Furthermore, THBS1 overexpression was shown to upregulate TGF-β1 and its downstream effectors Smad2/3 in MDCK cells, thereby promoting apoptosis, whereas THBS1 knockdown inhibited this pathway and enhanced proliferation [35]. Our findings are consistent with these previous reports. THBS1 overexpression in KGN cells promoted apoptosis, reduced proliferative activity, induced aberrant expression of inflammatory and steroidogenic genes, and activated the TGF-β/Smad pathway. Importantly, the TGF-β receptor inhibitor SB-431542 reversed THBS1-mediated GC dysfunction, confirming that the TGF-β/Smad pathway is a key downstream mediator of THBS1 action in PCOS. An alternative or complementary mechanism that has been proposed is the PI3K/AKT pathway: Zhang et al. [14] reported that the THBS1-derived tetrapeptide LSKL mitigates DHEA-induced apoptosis and oxidative stress via the THBS1/PI3K/AKT axis in rat granulosa cells. Our results, in which TGF-β receptor inhibition with SB-431542 was sufficient to rescue the major THBS1-induced phenotypes (apoptosis, inflammatory cytokines, steroidogenic dysregulation), support a TGF-β/Smad axis as a major contributor in our model system. We therefore consider TGF-β/Smad and PI3K/AKT not as mutually exclusive alternatives but as parallel branches of a THBS1-driven regulatory network whose relative weight may depend on the cell context and the form of THBS1 implicated (full-length matricellular protein versus cleavage-derived peptides such as LSKL). A formal dissection of the relative contribution of each branch would require Smad2/3 silencing and rescue with kinase-dead AKT mutants, both of which we identify as priorities for future work.

Despite the agreement among the patient, animal and cellular data, several limitations qualify the conclusions of this study. (1) The clinical cohort is single-centre with 21 patients per arm; THBS1 should therefore be interpreted as a candidate biomarker and exploratory therapeutic target pending validation in independent multicentre cohorts with formal ROC analysis and a pre-specified validation set. (2) The KGN cell line is widely used (>500 PCOS-related publications) but is granulosa-tumour derived; validation of the key transcriptional and apoptotic findings in primary human granulosa cells from independent donors remains an important next step. (3) The DHEA-induced PCOS rat model recapitulates reproductive features but only partially captures the metabolic dimensions of human PCOS; comprehensive metabolic profiling (oral glucose tolerance test, insulin tolerance test) and ovarian fibrosis quantification (Masson’s trichrome, α-SMA, collagen-I) are indicated for dedicated longitudinal studies. (4) Pharmacological TGF-β receptor inhibition (SB-431542) targets the same TGFβR1 → Smad2/3 phosphorylation node as genetic Smad2/3 ablation, but a formal genetic-rescue experiment (siSmad2/3 or dominant-negative constructs) remains to be performed. (5) Although co-immunoprecipitation, the latent TGF-β1 docking model and the active/total TGF-β1 ELISA together support functional THBS1–TGF-β1 interaction, kinetic binding parameters by surface plasmon resonance or biolayer interferometry would provide additional rigour. (6) Ovarian tissue homogenate active/total TGF-β1 measurement was not feasible because tissue samples were exhausted; serum and follicular fluid ELISA are reported as the closest available proxies. We thus consider this study an exploratory pilot whose hypotheses warrant validation in larger and methodologically broader follow-up work.

In summary, this study elucidates a mechanism by which THBS1 exacerbates ovarian granulosa cell dysfunction in PCOS via activation of the TGF-β/Smad signaling pathway. Further investigation into the translational potential of targeting THBS1 may facilitate the development of novel therapeutic strategies for women with PCOS.

## 4. Materials and Methods

### 4.1. Patients and Tissue Samples

This study enrolled PCOS patients who underwent IVF/ICSI-ET treatment at the First Hospital of Lanzhou University from June to December 2021, as well as age-matched control subjects. Follicular fluid was collected on the oocyte retrieval day and centrifuged; the supernatant was stored at −80 °C, and granulosa cells were extracted from the pellet and preserved at −80 °C. Patients with a history of hormone medication, ovarian surgery, or endocrine disorders were excluded. This study was approved by the Ethics Committee (LDYYLL2021-215), and informed consent was obtained from all participants.

### 4.2. Animal Model

Twenty 3-week-old female Sprague-Dawley rats (Lanzhou University Animal Centre, certificate SCXK [Gan] 2022-0006) were randomly allocated to four groups (*n* = 5): Control, DHEA, DHEA + AAV9-Ctrl, and DHEA + AAV9-THBS1. Control rats received daily subcutaneous injection of olive oil; DHEA, DHEA + AAV9-Ctrl and DHEA + AAV9-THBS1 groups received DHEA (6 mg per 100 g body weight, dissolved in olive oil, Sigma D4000) subcutaneously for 28 consecutive days. On day 6, rats were anaesthetised with isoflurane, and a dorsal skin incision was made to expose both ovaries. AAV9 vector (5 μL per injection site, three sites per ovary, delivering 7.5 × 10^10^ viral genomes per ovary; AAV9-THBS1 titre 7.98 × 10^12^ VG·mL^−1^, AAV9-Ctrl titre 6.25 × 10^12^ VG·mL^−1^; Shanghai Genechem, Shanghai, China) was injected subcapsularly into both ovaries. After the procedure, the incision was sutured. Estrous cycles were monitored daily by vaginal smear cytology from days 13–28. On day 29, rats were euthanized by intraperitoneal injection of a high-concentration (200 mg/mL) sodium pentobarbital solution combined with lidocaine; serum and ovaries were harvested for downstream assays. All animal procedures were approved by the Animal Ethics Committee of the First Hospital of Lanzhou University (Approval No. LDYYLL2025-798).

### 4.3. Cell Culture

The human ovarian granulosa tumor cell line KGN was purchased from Procell Life Science & Technology Co., Ltd. (Wuhan, China). Cells were maintained in DMEM/F12 medium supplemented with 10% fetal bovine serum (FBS) and 1% penicillin/streptomycin (NCM Biotech, Suzhou, China) in a humidified incubator at 37 °C with 5% CO_2_. The cell line was authenticated by short tandem repeat (STR) profiling and routinely confirmed to be free of mycoplasma contamination by qPCR-based detection.

### 4.4. DHEA, SB-431542 and AAV-9

The DHEA oil solution was prepared by dissolving 120 mg DHEA powder in 200 μL absolute ethanol, followed by the addition of 10 mL olive oil and thorough shaking to achieve a final concentration of 12 mg/mL. SB-431542 (Cat. No. HY-10431, MedChemExpress, Monmouth Junction, NJ, USA) was prepared as follows: for the 10 mM stock solution, the powder was removed from the refrigerator and equilibrated at room temperature for 10–15 min; 1 mg of powder was accurately weighed using an analytical balance and transferred to a 1.5 mL enzyme-free centrifuge tube, to which 260 μL DMSO was added, followed by vortexing for 2 min to ensure complete dissolution. For the 100 μM working solution, 1 μL of the 10 mM stock solution was mixed with 99 μL ddH_2_O. All stock solutions were aliquoted and stored at −80 °C. Working solutions were freshly prepared on the day of use.

RNAi technology was used to knock down the rat Thbs1 gene. RNAi target sequences were designed against Thbs1 (Gene ID: 445442) and cloned into the GV478 vector (U6-MCS-CAG-EGFP) to construct recombinant adeno-associated virus (serotype 9). The construct was confirmed by sequencing, and the viruses were packaged with titers were 7.98 × 10^12^ VG·mL^−1^. The control virus contained the insertion sequence CGCTGAGTACTTCGAAATGTC. The viruses were prepared by Shanghai Genechem Co., Ltd. (Shanghai, China).

### 4.5. Plasmid Construction and Transfection

KGN cells were seeded at a density of 3 × 10^5^ cells per well in 6-well plates and transfected when reaching 70–80% confluence. A total of 4 μg plasmid and 5 μL Lipofectamine 2000 were separately diluted in 250 μL Opti-MEM, mixed and incubated for 15 min before dropwise addition to the cells. The medium was replaced with complete culture medium after 6 h, and cells were harvested for RNA or protein extraction 48 h post-transfection.

### 4.6. RNA Sequencing

Total RNA was extracted from KGN cells 48 h after transient transfection with an empty pcDNA3.1 plasmid (n = 3) or pcDNA3.1-THBS1 (n = 3) using TRIzol (Invitrogen, Carlsbad, CA, USA). RNA quality (RIN ≥ 8.0) was confirmed on an Agilent 2100 Bioanalyzer(Agilent Technologies, Santa Clara, CA, USA). Strand-specific paired-end (150 bp) libraries were prepared with the NEBNext Ultra II Directional RNA Library Prep Kit and sequenced on an Illumina NovaSeq 6000 platform by Shanghai Majorbio Bio-Pharm Technology (Shanghai, China), yielding 5.4–6.6 Gb (37–43 million paired-end reads) per sample with Q30 ≥ 95.7%. Reads were quality-trimmed with fastp v0.23.4, aligned to the GRCh38/Ensembl release 110 reference genome with HISAT2 v2.2.1 (unique mapping rate 93.7–94.2%), assembled with StringTie v2.2.1, and quantified with RSEM v1.3.3. Differential expression analysis was performed with DESeq2 v1.42.0; genes with Benjamini–Hochberg-adjusted *p* < 0.05 and |log_2_FC| > 1 were defined as differentially expressed. Pathway analyses comprised over-representation analysis with Enrichr (KEGG_2021_Human) and Gene Set Enrichment Analysis with gseapy v1.2 (MSigDB Hallmark v2024, KEGG, Reactome) using a signed rank metric of sign (log_2_FC) × −log_10_(P). Sample identity was verified by THBS1 expression and cross-checked with in-house qPCR validation; one tube-labelling discrepancy was identified after sequencing and corrected in the final metadata before downstream analysis. All raw and processed data have been deposited at the Gene Expression Omnibus (accession [GSE330821]).

### 4.7. Granulosa Cell Identification

Granulosa cells were resuspended in PBS, smeared onto glass slides, and air-dried at 4 °C. After fixation with immunostaining fixative at room temperature for 15 min and permeabilization with 0.5% Triton X-100 for 10 min, cells were incubated with FSHR primary antibody (1:200) overnight at 4 °C, followed by Coralite594-conjugated secondary antibody for 2 h at room temperature in the dark. Nuclei were stained with DAPI for 10 min. Samples were mounted and observed under a laser confocal microscope.

### 4.8. Cell Proliferation Assay

KGN cells from different treatment groups were seeded at 5 × 10^3^ cells per well in 96-well plates and incubated overnight. Cell Counting Kit-8 (CCK-8) reagent (Coolaber, Beijing, China) was added according to the manufacturer’s instructions. After further incubation for 2 h at 0, 24, 48, and 72 h post-seeding, absorbance at 450 nm was measured using a microplate reader (TECAN, Nänikon, Switzerland).

Cell viability (%) = [(OD_treatment/OD_blank)/(OD_control/OD_blank)] × 100

### 4.9. Quantitative Real-Time PCR

Total RNA was extracted using the RNA-Quick Purification Kit (ESscience, China), and reverse transcription was performed with the PrimeScript RT Reagent Kit (Accurate Biology, China). qPCR was conducted using Accurate Taq Master Mix (Accurate Biology, China) on the LightCycler 480 (Roche Diagnostics). The thermocycling conditions were as follows: initial denaturation at 95 °C for 30 s, followed by 40 cycles of 95 °C for 5 s and 60 °C for 30 s. A melt-curve analysis was performed after amplification to verify product specificity. Gene expression was calculated using the 2^(−ΔΔCt)^ method and normalized to GAPDH. Primers were designed to span exon–exon junctions where possible, ensuring specificity. All qPCR primers are listed in Table 2.

### 4.10. Enzyme-Linked Immunosorbent Assay (ELISA)

Standards were prepared according to the ELISA kit instructions. Blank wells, standard wells (50 μL standard), and sample wells (40 μL diluent + 10 μL follicular fluid, triplicates per group) were set up and incubated at 37 °C for 30 min, followed by five washes. Enzyme conjugate (50 μL) was added to all wells except blanks and incubated at 37 °C for 30 min, followed by another five washes. Chromogen A and B (50 μL each) were added, and the plate was incubated at 37 °C in the dark for 10 min. Stop solution (50 μL) was added, and absorbance at 450 nm was measured. Sample concentrations were calculated from the standard curve and multiplied by the dilution factor to obtain actual concentrations.

### 4.11. Western Blot

Proteins were extracted from proliferating KGN cells using RIPA lysis buffer supplemented with protease and phosphatase inhibitors. Protein concentration was determined with the BCA Protein Assay Kit (Boster, Wuhan, China). Equal amounts of denatured proteins were separated by 10% SDS-PAGE and then transferred onto polyvinylidene fluoride membranes (Millipore, Billerica, MA, USA) using a semi-dry transfer system at 25 V for 30 min. Membranes were blocked with 5% skim milk in Tris-buffered saline containing 0.1% Tween-20 (TBS-T) for 1 h at room temperature. Following blocking, membranes were incubated overnight at 4 °C with primary antibodies (all at 1:1000 dilution): rabbit anti-β-actin (81115-1-RR, Proteintech, Singapore), mouse anti-GAPDH (60004-1-Ig, Proteintech), rabbit anti-THBS1 (ab263905, Abcam, Cambridge, UK), rabbit anti-BAX (50599-2-Ig, Proteintech), rabbit anti-Bcl-2 (A19693, ABclonal, Woburn, MA, USA), rabbit anti-IL-6 (A22222, ABclonal), rabbit anti-TNF-α (A22227, ABclonal), rabbit anti-CYP17A1 (A1373, ABclonal), rabbit anti-CYP19A1 (A12238, ABclonal), rabbit anti-SMAD2/3 (A28421, ABclonal), rabbit anti-P-SMAD2/3 (AP1343, ABclonal), and rabbit anti-TGFB1 (10188-1-AP, Proteintech). After washing, membranes were incubated with horseradish peroxidase (HRP)-conjugated secondary antibodies (1:5000 dilution) for 1 h at room temperature. Protein bands were visualized using an enhanced chemiluminescence (ECL) substrate (Bio-Rad, Hercules, CA, USA) and imaged with a ChemiDoc imaging system (Bio-Rad). Densitometric analysis was performed using ImageJ software, and target protein expression levels were normalized to the corresponding loading control (β-actin or GAPDH).

Antibodies and reagents used in this study, together with manufacturer and catalogue numbers, are listed in Table S1. Antibody specificity was previously validated by the manufacturer; refer to product datasheets for the corresponding KO/KD or peptide-competition validation evidence.

### 4.12. Co-Immunoprecipitation (Co-IP)

Cells were collected and whole-cell lysates were prepared and incubated with the appropriate primary antibody overnight at 4 °C. The lysates were washed three times with lysis buffer, followed by the addition of magnetic beads and incubation at room temperature for 1 h. After three additional washes, the immunoprecipitated protein complexes were boiled and subjected to Western blot analysis.

### 4.13. Immunofluorescence (IF)

Following in vitro treatment, cells were fixed with 4% paraformaldehyde for 15 min at room temperature. Cells were then permeabilized with 0.2% Triton X-100 for 10 min at room temperature. After washing, cells were blocked with 5% bovine serum albumin (BSA) in phosphate-buffered saline (PBS) for 1 h at room temperature. Subsequently, cells were incubated overnight at 4 °C with the primary antibody: rabbit anti-FSHR (Servicebio, Wuhan, China, GB11275-1, 1:200 dilution). After washing, cells were incubated with the secondary antibody, Alexa Fluor 594-conjugated goat anti-rabbit IgG (1:500 dilution), for 1 h at room temperature in the dark. Nuclei were counterstained with DAPI at a concentration of 1 µg·mL^−1^ for 10 min at room temperature. Finally, slides were mounted with an antifade mounting medium. Fluorescence images were acquired using a Leica TCS SP8 confocal microscope equipped with a ×63 oil immersion objective and 405 nm (DAPI) and 561 nm (Alexa Fluor 594) lasers.

### 4.14. Hematoxylin and Eosin (H&E) Staining

Paraffin sections were sequentially dewaxed in xylene I and II for 20 min each, rehydrated through absolute ethanol, 75% ethanol and tap water for 5 min each, and stained with hematoxylin (Servicebio G1004) for 5 min. After rinsing under running tap water, sections were differentiated with 1% hydrochloric-acid ethanol differentiation solution (Servicebio G1039) for 5 s, blued with Scott’s tap-water-substitute bluing solution (Servicebio G1040) for 10 s, dehydrated through 85% and 95% ethanol (5 min each), and stained with eosin (Servicebio G1001) for 20 s. Sections were rinsed in tap water for 1 min, dehydrated through absolute ethanol I–III and xylene I–II (5 min each) and mounted in neutral balsam (Servicebio G1403). Light-microscopic examination showed blue nuclei and red cytoplasm, with no precipitates.

### 4.15. Immunohistochemistry (IHC)

Immunohistochemistry was performed on 4-μm paraffin-embedded ovarian sections from each of the four rat groups. Sections were baked at 65 °C for 1 h to improve adhesion, deparaffinised in xylene and rehydrated through a graded ethanol series. Antigen retrieval was performed by heat-induced epitope retrieval in citrate buffer pH 6.0 at 95 °C for 20 min, followed by cooling to room temperature. Endogenous peroxidase activity was quenched with 3% H_2_O_2_ for 10 min, and non-specific binding was blocked with 5% goat serum (Servicebio G1208) for 1 h at room temperature. Sections were incubated with primary antibody overnight at 4 °C: rabbit anti-THBS1 (Proteintech 18304-1-AP, 1:100) or rabbit anti-IL-6 (Servicebio GB11117, 1:800). The next day, sections were washed in PBS and incubated with HRP-polymer anti-rabbit IgG (Servicebio G1213, ready-to-use) for 30 min at room temperature. Bound antibody was visualised with 3,3′-diaminobenzidine (DAB, Servicebio G1212) chromogen for 1–3 min under microscopic control, counterstained with hematoxylin (Servicebio G1004, 10 s), dehydrated and mounted in neutral balsam (Servicebio G1403). Whole-slide images were captured on a Pannoramic SCAN II digital scanner (3DHISTECH, Budapest, Hungary); positive staining was quantified as mean optical density (MOD) in three independent fields per ovary using ImageJ v1.53 (NIH) with the IHC Profiler plug-in. Antibody specificity was previously validated by the manufacturer (KO/KD or peptide-blocking validation evidence is provided on the product datasheet).

### 4.16. Cell Cycle Assay

Cell cycle distribution was analyzed using the Cell Cycle Staining Kit (MultiSciences CCS012, Hangzhou, China). At 48 h post-transfection, cells were collected, washed with PBS, digested with trypsin (EDTA-free, NCM Biotech, Suzhou, China), and terminated. After two washes with 1× PBS, cells were centrifuged and the supernatant was discarded. Cells were washed again with ice-cold PBS, centrifuged, and resuspended in DNA Staining solution and Permeabilization buffer by vortexing. After incubation at room temperature in the dark for 30 min, samples were analyzed by flow cytometry.

### 4.17. Apoptosis Detection

Apoptosis was detected using the Annexin V-FITC/PI Apoptosis Detection Kit (MultiSciences AP101, Hangzhou, China). Cells were collected with trypsin (EDTA-free, NCM Biotech, Suzhou, China) and washed twice with 1× PBS. A total of 1 × 10^5^ cells were resuspended in 500 μL binding buffer, followed by the addition of 5 μL Annexin V-FITC and 10 μL PI. After incubation at room temperature in the dark for 15 min, apoptosis was immediately analyzed by flow cytometry (BD, Franklin Lakes, NJ, USA). Stained cells were classified into three groups: viable cells (double negative, Q4), apoptotic cells (Annexin V-FITC positive, Q2 + Q3), and necrotic cells (Annexin V-FITC negative/PI positive, Q1).

### 4.18. Molecular Docking

The crystal structure of human latent TGF-β1 (PDB 9VJJ, deposited 20 June 2025; Mol_ID 1, chains A and B comprising the LAP domain and the mature TGF-β1 cytokine) was retrieved from the Protein Data Bank. The co-crystallised SOF10 Fab (chains H, I, L, M; molecular IDs 2 and 3) was removed, and remaining waters and crystal additives were stripped. The human THBS1 receptor model was downloaded from UniProt (entry P07996) and energy-minimised in the GROMOS96 43B1 force field (SwissPDB-Viewer v4.10). Protein–protein docking was performed using HDOCKlite v1.1 (default settings, 2000 sampled binding modes) with the THBS1 model as the receptor and chain A of 9VJJ as the ligand. Binding free energies of the ten top-ranked complexes were further evaluated by MM/GBSA on the HawkDock server. The interface of the highest-scoring model was analysed using the PLIP web server with default parameters; hydrogen bonds (≤3.5 Å) and salt bridges (≤5.5 Å) were enumerated. Three independent docking runs were performed, and the top-ranked model was reproducible across runs.

### 4.19. Molecular Dynamics Simulation

The top-ranked docking complex was solvated in a TIP3P water cubic box with 1.0 nm padding and neutralised with Na^+^/Cl^−^ to 0.15 M ionic strength. The protein was modelled with the AMBER14SB force field. Long-range electrostatics were treated with the PME method (1.0 nm cutoff), and covalent bonds to hydrogen were constrained with LINCS. The system was energy-minimised (steepest descent, 50,000 steps), equilibrated at 310 K (NVT) and 1 bar (NPT) for 100 ps each, and then propagated under NPT conditions for 100 ns with a 2-fs time step using GROMACS 2023.2. Three independent replicates with different random seeds were performed; representative trajectories are shown. Trajectory analyses (RMSD, RMSF, Rg, SASA, inter-protein hydrogen bonds, free-energy landscape from PCA-projected RMSD/Rg) were performed with built-in GROMACS tools, and MM/PBSA binding free-energy decomposition was performed with the gmx_MMPBSA package v1.6 over the 60–100 ns equilibrated portion of the trajectory.

### 4.20. AAV Injection

Anaesthetised rats (5% isoflurane induction, 2% maintenance) were positioned in dorsal recumbency. After abdominal hair removal and skin disinfection, a 1.5-cm midline incision was made to expose both ovaries. A total of 5 μL recombinant AAV9 vector (AAV9-THBS1 titre 7.98 × 10^12^ VG·mL^−1^; AAV9-Ctrl titre 6.25 × 10^12^ VG·mL^−1^; Shanghai Genechem, Shanghai, China) was injected subcapsularly at three sites per ovary (≈1.5 μL per site), delivering 7.5 × 10^10^ viral genomes per ovary. The peritoneum and skin were closed in two layers and disinfected. Knockdown efficiency was verified by Western blot densitometry (ImageJ v1.53) at the experimental endpoint: ovarian THBS1 protein in DHEA + AAV9-THBS1 rats was reduced by 37.4% relative to DHEA + AAV9-Ctrl (*p* = 0.006, one-way ANOVA followed by Tukey HSD test, n = 3 per group), restoring it to a level statistically indistinguishable from the untreated Control group.

### 4.21. ELISA for Active and Total TGF-β1

Active and total TGF-β1 were quantified by sandwich ELISA (Fankew TGF-β1 kit) in (i) follicular fluid supernatants stored at −80 °C from the 21 PCOS patients and 21 age-matched controls described in Section 4.1, and (ii) serum collected on day 29 from the four animal groups. Each sample was assayed in two parallel reactions: “active” TGF-β1 was measured directly without sample pre-treatment; “total” TGF-β1 was measured after acid activation (1 N HCl, 10 min at room temperature, neutralised with 1.2 N NaOH/0.5 M HEPES). The latent fraction was calculated as latent = total − active. Standard curves were prepared in triplicate per kit instructions, and absorbance at 450 nm was measured on a TECAN microplate reader.

### 4.22. Statistical Analysis

Data were analysed in GraphPad Prism v9.0.1 and Python 3.11 with SciPy 1.17. Normality of distributions was tested by the Shapiro–Wilk test; homogeneity of variances by Levene’s test. Two-group comparisons used Student’s *t*-test (two-tailed) for normally distributed data or the Mann–Whitney U test otherwise. Multi-group comparisons used one-way or two-way ANOVA followed by Sidak’s or Tukey’s post hoc test. Correlations were assessed by Pearson’s r when both variables were normally distributed and Spearman’s ρ otherwise; correlation *p*-values were corrected for multiple testing using the Benjamini–Hochberg false-discovery-rate (FDR) method (the Bonferroni alternative is reported in parentheses for transparency). After FDR correction across the two pre-specified correlations, the THBS1–AMH correlation remained significant (Pearson r = 0.351, raw *p*= 0.036, *p*_FDR = 0.036; Bonferroni *p*_corr = 0.072) and the THBS1–LH correlation remained highly significant (Pearson r = 0.496, raw *p* = 0.0021, *p*_FDR = *p*_Bonf = 0.0042). Post hoc power analyses using statsmodels v0.14 returned an estimated power > 0.999 at α = 0.05 for the primary clinical comparison (AMH, Cohen’s d = 2.45) and 0.93 for the THBS1–LH correlation (r = 0.496, n = 42). All in vitro experiments were performed with three biological replicates (independent transfections/passages); all rat experiments used five biological replicates (animals) per group. Data are reported as mean ± standard error of the mean (SEM) unless otherwise stated. A two-sided *p*-value < 0.05 was considered significant unless stated. Exact *p*-values are reported in figure legends.

## Figures and Tables

**Table 1:** Comparison of clinical characteristics between the PCOS and Control groups.

Characteristics	Control (*n* = 21)	PCOS (*n* = 21)	*p*-Value
Age (years)	31.0 (29.0, 32.0)	29.0 (26.0, 31.0)	0.107
BMI (kg/m^2^)	21.60 ± 2.15	23.34 ± 3.48	0.097
Duration of infertility (y)	3.0 (1.5, 4.0)	3.0 (1.0, 4.0)	0.500
Primary infertility rate, *n* (%)	10 (47.62)	15 (71.42)	0.116
AFC (*n*)	13.90 ± 4.73	27.00 ± 8.00	0.018
AMH (ng/mL)	2.43 ± 1.29	10.32 ± 4.36	<0.001
Basal FSH (mIU/mL)	7.15 ± 1.48	5.51 ± 1.32	0.65
Basal LH (mIU/mL)	3.8 (3.4, 4.3)	8.1 (5.0, 11.8)	<0.001
Basal E2 (pg/mL)	42.1 (25.6, 49.6)	32.2 (22.9, 34.3)	0.050
Basal T (ng/dL)	12.20 (7.60, 23.40)	21.70 (18.70, 33.00)	0.003
Basal PRL (ng/mL)	18.3 (16.8, 25.6)	21.0 (14.2, 30.0)	0.263
TSH (mIU/L)	2.58 (1.97, 3.61)	3.10 (2.15, 3.83)	0.385
CA125 (U/mL)	15.2 (10.7, 25.9)	14.1 (9.9, 17.9)	0.497
TG (mmol/L)	1.30 (1.00, 1.90)	1.28 (0.90, 2.53)	0.563
TC (mmol/L)	4.39 ± 0.97	4.58 ± 0.74	0.156
LDL-C (mmol/L)	2.67 ± 0.72	2.89 ± 0.57	0.144
HDL-C (mmol/L)	1.48 (1.28, 1.63)	1.34 (1.24, 1.44)	0.204
HOMA-IR	1.10 (0.99, 1.65)	1.82 (1.18, 2.35)	0.038
Total gonadotropin days	10.0 (9.0, 12.0)	9.0 (8.0, 11.0)	0.150
Total gonadotropin dose (IU)	2475.0 (2025.0, 3050.0)	1650.0 (1312.5, 2062.5)	0.001
E2 on the day of HCG	3270.0 (1817.0, 5981.0)	6173.0 (4165.0, 9281.0)	0.002
P on the day of HCG	0.89 (0.58, 1.13)	0.94 (0.76, 1.35)	0.481
>16 mm follicles on the day of HCG (*n*)	13.0 (9.0, 18.0)	23.0 (20.0, 25.0)	<0.001
No. of retrieved oocytes (*n*)	14.28 ± 6.08	25.62 ± 8.73	0.061
No. of mature oocytes (*n*)	11.0 (8.0, 14.0)	25.0 (14.0, 26.0)	0.003
No. of available embryos (*n*)	8.38 ± 5.25	14.38 ± 7.67	0.173

BMI: Body mass index, AMH: Anti-Müllerian hormone, AFC: Antral follicular count, FSH: Follicle-stimulating hormone, LH: Luteinizing hormone, E2: Estradiol, T: Testosterone, PRL: Prolactin, TSH: Thyroid-Stimulating hormone, TG: Triglycerides, TC: Total cholesterol, LDL-C: Low-density lipoprotein, HDL-C: High-density lipoprotein, HOMA-IR: Insulin resistance index.

**Table 2:** The primer sequences used for RT-qPCR.

**Gene**	**Forward**	**Reverse**
*THBS1*	AGTTTGGAGGCAAGGACTGC	AGCTAGTACACTTCACGCCG
*IL6*	GAGGATTGTGGCCTTCTTTG	ACAGTTCCACAAAGGCATCC
*GAPDH*	CGAAGGTGGAGTCAACGGATTT	ATGGGTGGAATCATATTGGAAC

## Data Availability

Data is contained within the article and Supplementary Material.

## Supplementary Materials

The following supporting information can be downloaded at https://www.mdpi.com/article/10.3390/biomedicines14061273/s1.

## Abbreviations

The following abbreviations are used in this manuscript:

AAV	Adeno-Associated Virus
AFC	antral follicle count
AMH	anti-Müllerian hormone
BMI	body mass index
DHEA	Dehydroepiandrosterone
E2	estradiol
FSH	follicle-stimulating hormone
GCs	granulosa cells
Gn	gonadotropin
GnRH	gonadotropin-releasing hormone
HCG	Human Chorionic Gonadotropin
ICSI	intracytoplasmic sperm injection
IL-6	Interleukin-6
IR	insulin resistance
IVF	in vitro fertilization
LH	luteinizing hormone
PCOS	Polycystic ovary syndrome
T	Testosterone
TGF-β	Transforming Growth Factor-β
THBS1	Thrombospondin-1
WB	Western blot
