# Supplementary material for: THBS1 Induces Dysfunction of Ovarian Granulosa Cells in Patients with Polycystic Ovary Syndrome by Activating the TGF-β/Smad Pathway
Source: Biomedicines. 2026 Jun 2;14(6):1273. doi: 10.3390/biomedicines14061273 (PMC13297039; doi:10.3390/biomedicines14061273)
Supplement: Supplementary file 1 [file biomedicines-14-01273-s001.zip › Table S1.pdf]

Table S1. Antibodies and ELISA kits used in this study.

Reagent	Catalogue no.	Manufacturer
**Rabbit anti-FSHR**	GB11275-1	Servicebio
**Rabbit anti-β-actin**	81115-1-RR	Proteintech
**Rabbit anti-β-tubulin**	10094-1-AP	Proteintech
**Mouse anti-GAPDH**	60004-1-Ig	Proteintech
**Rabbit anti-THBS1 (Western blot/ Co-IP)**	ab263905	Abcam
**Rabbit anti-THBS1 (immunohistochemistry)**	18304-1-AP	Proteintech
**Rabbit anti-BAX**	50599-2-Ig	Proteintech
**Rabbit anti-Bcl-2**	A19693	ABclonal
**Rabbit anti-IL-6 (Western blot)**	A22222	ABclonal
**Rabbit anti-IL-6 (immunohistochemistry)**	GB11117	Servicebio
**Rabbit anti-TNF-α**	A22227	ABclonal
**Rabbit anti-CYP17A1**	A1373	ABclonal
**Rabbit anti-CYP19A1**	A12238	ABclonal
**Rabbit anti-SMAD2**	A19114	ABclonal
**Rabbit anti-phospho-SMAD2**	AP0269	ABclonal
**Rabbit anti-TGF-β1 (Western blot / Co-IP)**	10188-1-AP	Proteintech
**Rat E2 ELISA kit**	F3450-A	Fankew
**Rat FSH ELISA kit**	F3441-A	Fankew
**Rat LH ELISA kit**	F3499-A	Fankew
**Rat T ELISA kit**	F3452-A	Fankew
**Rat AMH ELISA kit**	F3095-A	Fankew
**Human THBS1 ELISA kit**	F10700-A	Fankew
**Human IL-6 ELISA kit**	F0049-A	Fankew
**Human TGF-β1 ELISA kit**	F0089-A	Multisciences/Fankew
**Rat TGF-β1 ELISA kit**	F3057-A	
