# Supplementary figures and images for: THBS1 Induces Dysfunction of Ovarian Granulosa Cells in Patients with Polycystic Ovary Syndrome by Activating the TGF-β/Smad Pathway
Source: Biomedicines. 2026 Jun 2;14(6):1273. doi: 10.3390/biomedicines14061273 (PMC13297039; doi:10.3390/biomedicines14061273)

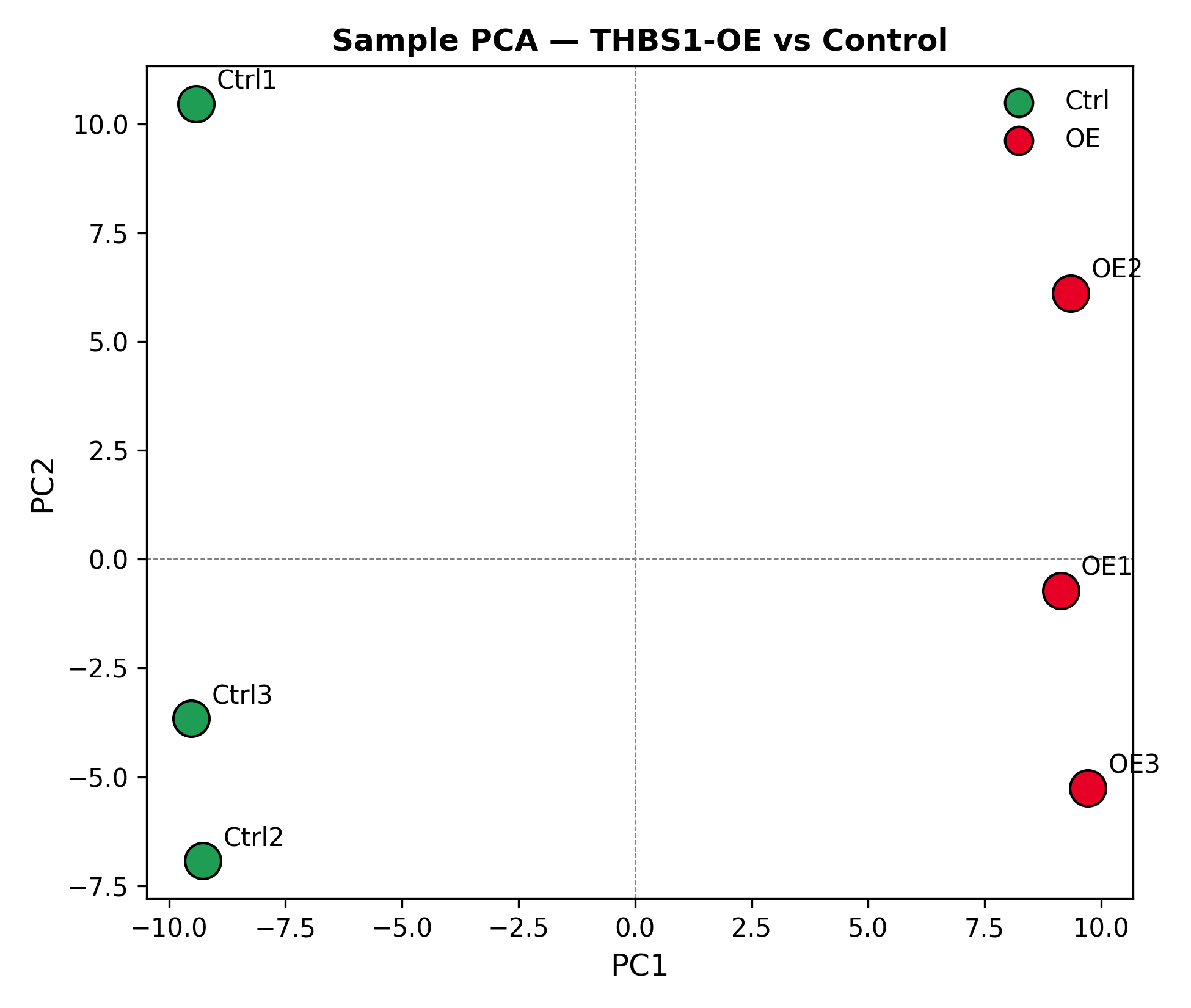

Supplement: Supplementary file 1 [file biomedicines-14-01273-s001.zip › FigS1_PCA.png]

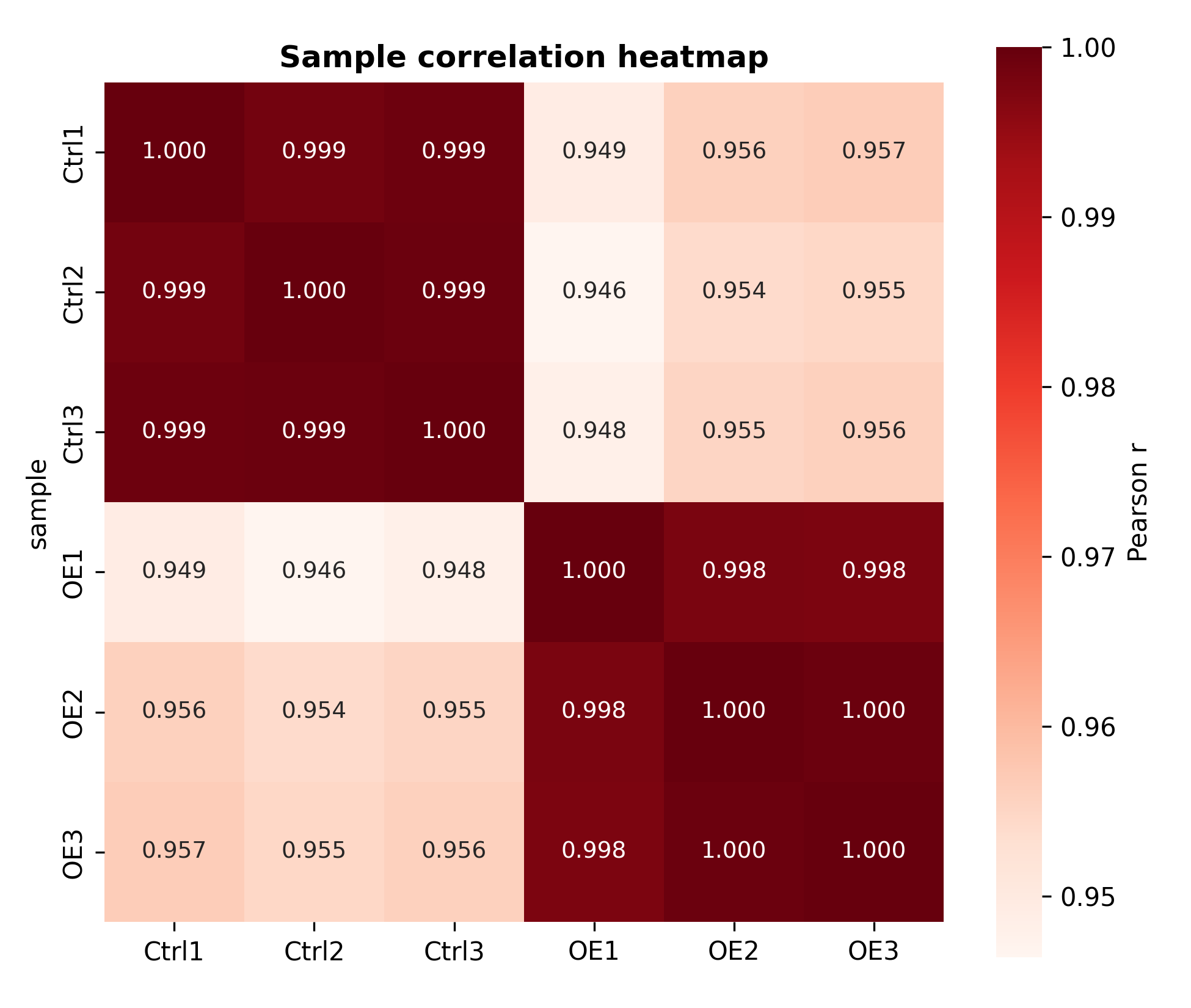

Supplement: Supplementary file 1 [file biomedicines-14-01273-s001.zip › FigS2_sample_correlation.png]
